# Antipsychotic-Related Hypothermia: Five New Cases

**DOI:** 10.3389/fpsyt.2019.00543

**Published:** 2019-07-29

**Authors:** Cherryl Zonnenberg, Jolien M. Bueno-de-Mesquita, Dharmindredew Ramlal, Jan Dirk Blom

**Affiliations:** ^1^Parnassia Academy, Parnassia Psychiatric Institute, The Hague, Netherlands; ^2^Faculty of Social Sciences, Leiden University, Leiden, Netherlands; ^3^Department of Psychiatry, University of Groningen, Groningen, Netherlands

**Keywords:** body temperature, neuroleptic, pharmacotherapy, psychosis, schizophrenia, side effect, thermoregulation

## Abstract

**Background:** Hypothermia is a potentially fatal adverse effect of antipsychotic drug (APD) use. With only 69 cases described in the literature, the condition is considered rare.

**Methods:** We describe five new cases, in which we estimated the role of clozapine, haloperidol, olanzapine, penfluridol, risperidone, and zuclopentixol with the aid of two structured assessment tools.

**Results:** In addition to APD use, all five patients described by us had been exposed to one or more additional predisposing factors for hypothermia. Therefore, with the aid of the assessment tools, the causal role of APDs was considered “possible” in four cases of moderate hypothermia and “doubtful” in the remaining one of mild hypothermia.

**Conclusion:** Although the best way to detect APD-related hypothermia is measuring the body temperature for a duration of at least 7–10 days after the start (or a dose increase) of APDs, the use of assessment tools to identify additional predisposing factors for hypothermia and to thus establish their causal relationship with APD use would seem to be valuable for clinical decision-making (i.e., whether or not to discontinue APD use). Further research is needed to obtain reliable prevalence figures for APD-related hypothermia and its consequences, preferably in relation with physiological changes in body temperature.

## Introduction

Four homeless people died of exposure to cold in Portland, Oregon, during the first 10 days of 2017, after temperatures had unexpectedly dropped to −7°C (1). One of the victims was a 52-year-old woman diagnosed with schizophrenia who had lost her home a few months beforehand. She was found in a parking garage, mumbling and confused, and in the act of paradoxically undressing herself, a well-known symptom of lethal hypothermia (2). Paramedics were called to the scene, but nonetheless, the woman died within half an hour. Her fate resonated with the local community and drew attention to the need for care for the homeless. Although various obvious predisposing factors for hypothermia had been present (i.e., outdoor exposure to cold and inadequate clothing), an additional factor may well have been the use of antipsychotic drugs (APDs).

APD-related hypothermia was first described by Loughnane (3). Although its underlying pathophysiological mechanism is not entirely clear, peripheral vasodilatation *and* a failure of central thermoregulation seem to play a role (4). In spite of its potentially fatal consequences, APD-related hypothermia has received substantially less attention than its clinical counterpart, APD-related *hyper*thermia, which has been described extensively in the context of malignant antipsychotic syndrome (5). For the purpose of a prior systematic review, we found no more than 57 original case descriptions of APD-related hypothermia, published over a time span of 50 years (i.e., 1.1 cases per year; 6). From these cases, we inferred that the risk of APD-related hypothermia is highest during the week following the initiation—or a dose increase—of APDs, notably in combination with old age, exposure to cold, the adjuvant use of benzodiazepines, and/or (subclinical) hypothyroidism. On the basis of data from drug-monitoring agencies, we moreover inferred that the prevalence of APD-related hypothermia may well be 10 times higher than that suggested by the literature (6). The publication of seven additional papers over the past 2 years, collectively describing 12 new cases, indicates that awareness of this clinically relevant condition may finally be growing (7–13). To add to the burgeoning body of literature, we here describe five cases from our own clinical practice and highlight implications for clinical practice.

## Methods

All five patients presented here were under our treatment at Parnassia Psychiatric Institute (The Hague). Patients B and D provided written permission for publication of their cases. On behalf of patients A, C, and E, written permission was obtained from their respective family members, in accordance with Dutch national guidelines. In conformity with Sund-Levander et al. (14), we defined a normal body temperature for men (measured rectally) as 36.7–37.5°C and for women as 36.8–37.1°C. Following Khasawneh et al. (15), we defined *hypothermia* as a core body temperature of <35.0°C, *mild hypothermia* as 33–35°C, *moderate hypothermia* as 28–33°C, and *severe hypothermia* as <28°C (measured rectally). In all cases, a regular digital thermometer was used. To assess and quantify the causal role of APDs in the mediation of hypothermia, we used the Adverse Drug Reaction (ADR) Probability Scale (16). For calculating the mean number of predisposing factors for hypothermia, we used the methodology developed by Brevik and Farver (17).

## Results

### Presentation of Cases

#### Patient A

Patient A was a 74-year-old, unmarried Dutch man, who resided in the nursing home that is part of our psychiatric hospital. He had been diagnosed years ago with schizophrenia. His medication consisted of a zuclopentixol depot of 200 mg/week, vitamin D, acetylsalicylic acid, pantoprazole, and simvastatin. By the time we saw him, he was also using doxycycline because he was feeling ill and had slightly elevated infection parameters. The sedimentation rate was 48 mm/h (N 2–20 mm/h), and the C-reactive protein was 19 mg/l (N 0–8 mg/l). Nonetheless, a focus for the infection could not be found. Patient A presented with a body temperature of 32.5°C (measured rectally) at our outpatient department after his depot had been administered earlier that day. At the time of administration, it had been 36.8°C (measured aurically). The physical examination showed a bradycardia of 58 BPM but no other abnormalities. Patient A was diagnosed with moderate hypothermia, possibly due to the use of zuclopentixol. He was gradually rewarmed with warm blankets. His vital signs, which were being monitored, remained stable. After several hours, his body temperature normalized to 37.3°C. Although his hypothermia did not recur, the zuclopentixol depot was postponed and administered a week later. This time, the body temperature remained normal. However, during the subsequent 2 years, our patient had three more episodes of hypothermia (with temperatures of 34.6°C, measured aurically, and 33.4°C and 32.4°C, measured rectally, respectively, once while using zuclopentixol, once while using haloperidol). On all occasions, he recovered after gradual rewarming.

#### Patient B

Patient B was a 27-year-old, unmarried, homeless French man, who experienced psychotic symptoms, probably in the context of schizophrenia. His antipsychotic medication consisted of a weekly penfluridol depot of 20 mg. Although he did not use any additional medication, he did use heroin and cannabis. He was referred to our psychiatric hospital with a body temperature of 32.0°C (measured rectally) after the police had found him wandering the streets. He had made a confused impression and had told the police officers that he had been sleeping outdoors the night before. The outside temperature that day had ranged from 11.3 to 18.4°C (mean, 15.1°C). The physical examination revealed a bradycardia of 47 BPM. The ECG showed pointed T-tops (which were probably idiopathic or due to hypokalemia) and signs of possible left-ventricle hypertrophy. His urine tested positive for heroin and cannabis. Patient B was diagnosed with moderate hypothermia, probably due to outdoor exposure to cold and the use of penfluridol (as well as heroin and cannabis). He was gradually rewarmed, and his vital signs were being monitored. Within a few hours, his body temperature normalized to 35.6°C. The penfluridol was discontinued. Since patient B refused to use APDs any longer, a period of 18 months elapsed before he could be persuaded to switch to haloperidol 3 mg/day. After that, he experienced no more episodes of hypothermia.

#### Patient C

Patient C was a 77-year-old, unmarried Dutch woman, who lived in the same nursing home as patient A. She had been diagnosed with a schizoaffective disorder and was treated with haloperidol 0.5 mg twice a day and lorazepam 8.5 mg/day. She had a history of transient ischemic attacks, cerebrovascular accidents, immobility, and subclinical hypothyroidism. She presented with a body temperature of 33.4°C (measured rectally). Her behavior was unaltered, there were no signs of illness, and her intake of food and drinks was normal. On that summer’s day, the weather had been warm, sunny, and dry but chilly at night (with a mean night temperature of 16.3°C). The physical examination revealed no additional abnormalities. Blood tests showed only signs of her subclinical hypothyroidism. Patient C was diagnosed with mild hypothermia, possibly due to the use of haloperidol. She was put to bed with warm blankets. However, the following morning, her temperature dropped even further to 32.8°C. She was once again rewarmed with the aid of warm blankets. Her vital signs were monitored, and this time her body temperature stabilized within a few hours. The haloperidol was continued, and, from then onwards, her body temperature was monitored closely. Four months later, while still on haloperidol, she developed a moderate hypothermia of 32.3°C (measured rectally). After gradual rewarming, her body temperature normalized to 36.7°C. One month later, patient C died, probably due to circulatory insufficiency in the context of dehydration, developed during a state of lethal catatonia.

#### Patient D

Patient D was a 65-year-old, divorced Hindustani-Surinamese man, who had been admitted to our psychiatric hospital for 9 months because of a psychotic relapse. He had previously been diagnosed with schizophrenia and was being treated with a haloperidol depot. Owing to severe extrapyramidal side effects, the haloperidol was switched to clozapine 50 mg/day. As his psychotic symptoms remained unaltered and the clozapine plasma level was 0.33 mg/l (N 0.35–0.80 mg/l), the dose was increased to 62.5 mg/day. Five days later, he presented with a Glasgow Coma Score of 6 (N 15) and a body temperature of 33.2°C (measured rectally). The physical examination showed a dry, flaky skin, and reduced skin turgor; there were no other abnormalities. Patient D had suffered from hypothyroidism in the past, but his hormone levels had been adequately restored with the aid of levothyroxine 0.025 mg/day. He was diagnosed with mild hypothermia, possibly due to the use of clozapine, and referred to a somatic hospital. There, a clozapine intoxication was excluded, and he was gradually rewarmed until his body temperature and consciousness had normalized. The clozapine was discontinued. With his psychotic symptoms untreated, patient D was unable to return to his home. As a consequence, he was referred to the nursing home of our psychiatric hospital. Four months later, he was diagnosed with active neurolues (i.e., neurosyphilis or tertiary syphilis), for which he was treated with benzathine benzylpenicillin. Because his psychotic symptoms did not subside, olanzapine 2.5 mg/day was added. The hypothermia did not recur.

#### Patient E

Patient E was a 59-year-old, married Dutch woman, who had resided for several years in the long-stay department of our psychiatric hospital. She had been diagnosed with a schizoaffective disorder. Owing to prior therapy resistance, she was treated with a combination of clozapine, risperidone, and olanzapine. The dosage of the risperidone was 9 mg/day (the other dosages are unknown). In addition, she also used lorazepam 7.5 mg/day. Because of a manic-psychotic relapse, the dose of risperidone was increased to 12 mg/day. Ten days later, she presented with a body temperature of 34.0°C (measured rectally). Her behavior was uncontrollable, and she was continually undressing. Although her intake of food and drinks had been adequate, she also suffered from mild renal failure, with a serum creatinine of 166 μmol/l (N 50–95 μmol/l) and a renal clearance of 27 ml/min (N > 52 ml/min). Despite a thrombocyte count of 24 × 10^9^/l (N 150–400 × 10^9^/l), there were no clinical signs of coagulopathy. Patient E was diagnosed with mild hypothermia, probably due to her unusual combination of APDs (especially the recent dose increase in risperidon) and her recurring state of undress. She was gradually rewarmed with the aid of warm blankets and warm drinks, under strict monitoring of her vital signs. The following day, clozapine and olanzapine were discontinued. Because the hypothermia persisted, 4 days later, the dosage of the risperidone was lowered to 6 mg/day. Additionally, amoxicillin/clavulanic acid was started in a dosage of 625 mg twice a day because of increased infection parameters [leukocyte count 11.4 × 10^9^/l (N 4.0–10.0 × 10^9^/l), neutrophil count 10.49 × 10^9^/l (N 1.5–7.5 × 10^9^/l), C-reactive protein 130 mg/l (N 0–8 mg/l)] in the absence of any clinical signs of infection. On day 6, her temperature normalized to 36.4°C, and the thrombocyte count also normalized. The antipsychotic treatment was continued with a combination of olanzapine 10 mg and aripiprazole 10 mg/day. Although hypothermia did not recur, patient E died three and a half years later, due to renal insufficiency.

### Summary and Analysis of the Case Series

All five patients described by us had been exposed to APDs, as well as to one or more additional predisposing factors for hypothermia (**Table 1**). With the aid of the methodology developed by Brevik and Farver (17), we calculated that the mean number of these factors was 2.6 (range, 2–4). The most prevalent factors were advanced age (60%) and (subclinical) hypothyroidism (40%). As quantified in accordance with the Adverse Drug Reaction (ADR) Probability Scale (16), in one case, there was a doubtful, and in four cases, a possible adverse drug-related event (**Table 2**).

**Table 1:** Degrees of hypothermia and analysis of predisposing factors.

Patient	Minimum body temperature (degree of hypothermia)	Type of antipsychotic	Number of additional predisposing factors (characterization)*
A	32.5°C (moderate)	Zuclopentixol	2 (PAH, MD)
B	32.0°C (moderate)	Penfluridol	2 (PAH, M)
C	32.3°C (moderate)	Haloperidol	4 (PAH, CNS, MD, M)
D	33.2°C (moderate)	Clozapine	2 (PAH, MD)
E	33.5°C (mild)	Risperidone Clozapine Olanzapine	3 (PAH, M, O)
			Mean: 2.6 (range 2–4)

*PAH, Primary Accidental Hypothermia; CNS, Central Nervous System; MD, Metabolic Disorder; M, Medication; O, other.

**Table 2:** Patient scores on the Naranjo Adverse Drug Reaction Probability Scale.

Patient (sex, age)	Score per episode of hypothermia*	Medication
A (M, 74 years)	4	Zuclopentixol
B (M, 27 years)	0	Penfluridol
C (F, 77 years)	4	Haloperidol
D (M, 65 years)	3	Clozapine
E (F, 59 years)	3 0 0	Risperidone Clozapine Olanzapine

*Scoring legend:

>9 = definitive adverse drug reaction.

5 to 8 = probable adverse drug reaction.

1 to 4 = possible adverse drug reaction.

0 = doubtful adverse drug reaction.

## Discussion

Contrary to the woman in Portland, none of our five patients died—at least not during a phase of hypothermia. This may well be due to the fact that none of them had been exposed to freezing, but perhaps also to the fact that they had all been under regular care, and that even patient B, the homeless man who had slept outside, had been detected in time by the police and referred to our hospital. Since systematic studies are lacking, the actual prevalence of APD-related hypothermia is unknown, as is the proportion of fatal outcomes. With APD use ranging worldwide from 3.2/1,000 in Colombia to 78.2/1,000 in Taiwan (18), the impact of ensuing hypothermia on global health must be substantial. In the present case series, we describe hypothermia in the context of clozapine, haloperidol, olanzapine, penfluridol, risperidone, and zuclopentixol use. A recent review by our group showed that APD-related hypothermia had been described before in the context of haloperidol (13 cases), clozapine (3 cases), risperidone (10 cases), and olanzapine (13 cases) use (6). As far as we know, there are no earlier publications of this type on penfluridol and zuclopentixol. Given the fact that undertreatment of psychosis has its own adverse effects on mental and physical health, quality of life, and mortality (19), the solution would not seem to lie in withholding psychotic patients from treatment with APDs, but rather in proper monitoring of the body temperature. This should be done for a duration of at least 7–10 days after starting with an APD or a dose increase (6), although some authors argue that several weeks would be even better, since hypothermia has also been described after two or more weeks (20, 21). That said, our case series indicates that even in the absence of any dose alterations, APD use may be a risk factor for hypothermia. This finding is all the more pressing, since individuals with psychosis are at risk for hypothermia anyway due to a lower mean baseline temperature and—if present—poverty and/or homelessness, which have also been established as risk factors (22–25). Although treatment of hypothermia is relatively easy and cheap (26), prevention would seem to be the solution worth striving for. If it has already set in, the goal must be early detection and treatment, preferably aided by an assessment of the causal role of APDs.

The body temperature should preferably be measured with the aid of a hypothermia thermometer. When frequent monitoring is not feasible, the method developed by Brevik and Farver (17) may be helpful to identify patients with an elevated risk of APD-related hypothermia. This method allows for a quick inventory of predisposing factors and a global impression of the severity of the ensuing risk. Still, the decision whether to continue or discontinue an APD may prove difficult. In clinical practice, this needs to be assessed in the light of the severity of the psychotic symptoms that might recur. Usually, cases of APD-related hypothermia tend to resolve—after proper monitoring and treatment—within 24–48 h. After that, only few patients tend to experience another episode of hypothermia, even after continuing the original APD or switching to another type (6). To facilitate the decision-making process, a structured assessment tool such as the ADR Probability Scale (16) may be of use, which gives an estimate of the causal role of APDs in the presence of other predisposing factors for hypothermia. If APD use was the only predisposing factor, and the causal role “possible,” “probable,” or “definitive” (**Table 2**), we recommend to stop or lower the dose during rewarming. If this is not possible, we advise to continue the APD under strict monitoring of the temperature and other vital functions. Finally, we recommend to review the APD treatment regimen *an sich* (e.g., proper dosage and—if possible—monotherapy). As may have been the case with our patient E, polypharmacy may add to the risk of APD-related hypothermia. After normalization of the temperature, there would seem to be no strict contraindications for reintroducing the same APD, even in its initial dose, as long as proper attention for additional predisposing factors for hypothermia is guaranteed (6).

## Conclusions

With the five new cases of APD-related hypothermia here described, we add to the burgeoning literature on this underreported and still poorly understood side effect. Moreover, as far as we know, this is the first description of hypothermia in the context of penfluridol and zuclopentixol. To prevent severe APD-related hypothermia and detect it at an early stage, we recommend to measure the body temperature for a duration of 7–10 days after starting—or increasing the dose of—APDs. When this is not possible, we recommend to estimate the risk of hypothermia while also considering the role of additional predisposing factors. In cases of established hypothermia, we recommend to estimate the causal role of APDs with the aid of a structured assessment tool, such as the Naranjo Adverse Drug Reaction Probability Scale, and use the outcome to guide clinical decision-making (i.e., whether to continue or discontinue this specific APD).

## Limitations

Our knowledge of APD-related hypothermia is still limited, as is our knowledge of the prevalence of idiopathic hypothermia in the general population. As a consequence, a reliable estimation of the relative risk of hypothermia due to APD use is hard to make.

## Data Availability

All datasets generated for this study are included in the manuscript and the supplementary files.

## Ethics Statement

The study was exempt from testing by a medical ethical committee. All five patients presented here were under our treatment at Parnassia Psychiatric Institute (The Hague). Patients B and D provided written permission for publication of their cases. On behalf of patients A, C, and E, written permission was obtained from their respective family members.

## Author contributions

CZ contributed to the conception and design of the work, and to the acquisition, analysis, and interpretation of data for the work, drafted and revised the work, gave final approval for the final version to be published, and agreed to be accountable for all aspects of the work in ensuring that questions related to the accuracy or integrity of any part of the work are appropriately investigated and resolved. JB-d-M contributed to the conception and design of the work, and to the acquisition, analysis, and interpretation of data for the work, revised the work, gave final approval for the final version to be published, and agreed to be accountable for all aspects of the work in ensuring that questions related to the accuracy or integrity of any part of the work are appropriately investigated and resolved. DR contributed to the interpretation of data for the work, revised the work, gave final approval for the final version to be published, and agreed to be accountable for all aspects of the work in ensuring that questions related to the accuracy or integrity of any part of the work are appropriately investigated and resolved. JDB contributed to the conception and design of the work, and to the analysis, and interpretation of data for the work, drafted and revised the work, gave final approval for the final version to be published, and agreed to be accountable for all aspects of the work in ensuring that questions related to the accuracy or integrity of any part of the work are appropriately investigated and resolved.

## Conflict of Interest Statement

The authors declare that the research was conducted in the absence of any commercial or financial relationships that could be construed as a potential conflict of interest**.**

## References

[1] Wilson J The death of Karen Batts: the homelessness case that shocked Portland. The Guardian (2017). https://www.theguardian.com/society/2017/feb/17/karen-batts-homelessness-death-portland-oregon Retrieved on January 20, 2019.

[2] Rothschild MA Lethal hypothermia: paradoxical undressing and hide-and-die syndrome can produce obscure death scene. Forensic Pathol Rev (2004) 1:263–72. 10.1007/978-1-59259-786-4_11

[3] Loughnane T Hypothermia in a young adult. Lancet (1968) 292:455–56. 10.1016/S0140-6736(68)90491-1 4174175

[4] Bueno de Mesquita J Balk FJE Olanzapine, hypothermie en winterse vrieskou. Psyfar (2013) 2:27–31.

[5] Tse L Barr AM Scarapicchia V Vila-Rodrigues F Neuroleptic malignant syndrome: a review from a clinically oriented perspective. Curr Neuropharmacol (2015) 13:395–406. 10.2174/1570159X13999150424113345 26411967 PMC4812801

[6] Zonnenberg C Bueno de Mesquita JM Ramlal D Blom JD Hypothermia due to antipsychotic medication: a systematic review. Front Psychiatry (2017) 8:165. 10.3389/fpsyt.2017.00165 28936184 PMC5594062

[7] Ajayi OO Holroyd S Severe recurrent hypothermia in an elderly patient with refractory mania associated with atypical antipsychotic, valproic acid and oxcarbazepine therapy. BMJ Case Rep (2017) bcr2017222462. 10.1136/bcr-2017-222462 PMC5720320 29197846

[8] Chen TR Chen YC Ouyang WC Zotepine-associated hypothermia in a schizophrenic patient. J Clin Psychopharmacol (2017) 37:367–8. 10.1097/JCP.0000000000000686 28277401

[9] Dekkers BGJ Eck RJ Maaten JC Touw DJ An acute oral intoxication with haloperidol decanoate. Am J Emerg Med (2017) 35:1387 e1–1387.e2. 10.1016/j.ajem.2017.07.013 28705742

[10] Käräjämäki A Lauri T Ebeling T Antipsychotic drug-induced hypoglycemia and hypothermia. Duodecim (2017) 133:301–4. 29205832

[11] Scherl TA Langguth B Kreuzer PM Hypothermia associated with antipsychotic medication: a clinical surveillance study. J Clin Psychopharmacol (2017) 37:751–3. 10.1097/JCP.0000000000000795 29045305

[12] Szota AM Araszkiewicz AS The risk factors, frequency and diagnosis of atypical drug-induced hypothermia: practical advice for doctors. Int Clin Psychopharmacol (2019) 34:1–8. 10.1097/YIC.0000000000000244 30398998

[13] Kozian R Schmid G Demian S Hypothermie unter der Gabe von Aripiprazol. Psychiatr Prax (2019) 46:49–51. 10.1055/a-0671-6803 30149396

[14] Sund-Levander M Forsberg C Wahren LK Normal oral, rectal, tympanic and axillary body temperature in adult men and women: a systematic literature review. Scand J Caring Sci (2002) 16:122–8. 10.1046/j.1471-6712.2002.00069.x 12000664

[15] Khasawneh FA Thomas A Thomas S Accidental hypothermia. Hospital Physician (2016) 12:16–21.

[16] Naranjo CA Busto U Sellers EM Sandor P Ruiz I Roberts EA A method for estimating the probability of adverse drug reactions. Clin Pharmacol Ther (1981) 30:239–45. 10.1038/clpt.1981.154 7249508

[17] Brevik A Farver D Atypical antipsychotic induced mild hypothermia. S D J Med (2003) 56:67–70. 12621956

[18] Hálfdánarson Ó Zoëga H Aagaard L Bernardo M Brandt L Fusté AC International trends in antipsychotic use: a study in 16 countries, 2005–2014. Eur Neuropsychopharmacol (2017) 27:1064–76. 10.1016/j.euroneuro.2017.07.001 28755801

[19] Sharifi V Eaton WW Wu LT Roth KB Burchett BM Mojtabai R Psychotic experiences and risk of death in the general population: 24–27 year follow-up of the Epidemiologic Catchment Area Study. Br J Psychiatry (2015) 207:30–6. 10.1192/bjp.bp.113.143198 PMC4486819 25953893

[20] Brandon Bookstaver P Miller AD Possible long-acting risperidone-induced hypothermia precipitating phenytoin toxicity in an elderly patient. J Clin Pharm Ther (2011) 36:426–42. 10.1111/j.1365-2710.2010.01189.x 21545623

[21] Kreuzer P Landgrebe M Wittmann M Schecklmann M Poppl TB Hajak G Hypothermia associated with antipsychotic drug use: a clinical case series and review of current literature. J Clin Psychopharmacol (2011) 52:1090–7. 10.1177/0091270011409233 21956608

[22] Irvine RE Hypothermia in old age. Practitioner (1974) 213:795–800. 4156586

[23] Shiloh R Hermesh H Weizer N Dorfman-Etrog P Weizman A Munitz H Acute antipsychotic drug administration lowers body temperature in drug-free male schizophrenic patients. Eur Neuropsychopharmacol (2000) 10:443–5. 10.1016/S0924-977X(00)00106-1 11115733

[24] Chong TWH Castle DJ Layer upon layer: thermoregulation in schizophrenia. Schizophr Res (2004) 69:149–57. 10.1016/S0920-9964(03)00222-6 15469188

[25] Kudoh A Takase H Takazawa T Chronic treatment with antipsychotics enhances intraoperative core hypothermia. Anesth Analg (2004) 98:111–5. 10.1213/01.ANE.0000093313.16711.5E 14693598

[26] Aslam AF Aslam AK Vasavada BC Khan IA Hypothermia: evaluation, electrocardiographic manifestations, and management. Am J Med (2006) 119:297–301. 10.1016/j.amjmed.2005.09.062 16564768

